# Trends in hospital admissions and clinical complexity in centenarians: a nationwide population-based study in Spain (2004–2020)

**DOI:** 10.1007/s41999-025-01362-1

**Published:** 2025-11-25

**Authors:** Juan Carlos Piñeiro-Fernández, Ramón Rabuñal-Rey, Eva Romay-Lema, Yelco Chantres-Legaspi, Ana María Santos-Martínez, Yoana Besteiro-Balado, Roi Suárez-Gil, Sonia Pértega-Díaz

**Affiliations:** 1 Department of Internal Medicine, Lucus Augusti University Hospital, SERGAS, 1 Ulises Romero Street, 27003 Lugo, Spain; 2 Infectious Diseases Unit. Lucus, Augusti University Hospital, SERGAS, 1 Ulises Romero Street, 27003 Lugo, Spain; 3 Rheumatology and Health Research Group, Department of Health Sciences, Faculty of Nursing and Podiatry, Universidade da Coruña, Esteiro, 15403 Ferrol, Spain; 4 Nursing and Health Care Research Group, Instituto de Investigación Biomédica de A Coruña (INIBIC), Xubias de Arriba 84, 15006 A Coruña, Spain

**Keywords:** Centenarians, Hospitalization, Comorbidity, Epidemiologic studies, Electronic Health Records

## Abstract

**Purpose:**

The aim of this study is to describe nationwide trends in clinical complexity, principal diagnoses, and in-hospital outcomes among centenarian hospital admissions in Spain, from 2004 to 2020.

**Methods:**

This study is a retrospective, population-based study using the Spanish National Hospital Discharge Database. All unplanned admissions of patients aged ≥ 100 years were analyzed across three periods (2004–2010, 2011–2015, 2016–2020). Trends in comorbidity, diagnoses, complications, mortality, and length of stay (LOS) were assessed using joinpoint regression models.

**Results:**

A total of 43,730 hospital admissions were analyzed. The prevalence of severe comorbidity rose from 17.6 to 31.8% (*p* < 0.001), and multimorbidity from 61.4 to 76.6% (*p* < 0.001), with parallel rises in dementia and other frailty-related conditions. Infections became the leading cause of admission (*p* < 0.001), particularly respiratory (from 18.4 to 26.1%), and urinary infections (from 4.2 to 7.9%). All types of in-hospital complications rose steadily, with an upward trend in patients with ≥ 3 complications, especially in the last 5 years (Annual percent changes (APC) = 14.9%; 95% confidence interval (CI) 9.7%; 20.4%) and among women (APC = 15.9%; 95% CI 10.7%; 21.5%). In-hospital mortality rose modestly (APC = 1.0%; 95% CI 0.5%; 1.6%), while average LOS decreased from 8.7 ± 10.5 to 7.3 ± 7.3 days (*p* < 0.001). Discharges to nursing homes increased from 2.1 to 4.8% (*p* < 0.001).

**Conclusion:**

Centenarians admitted to Spanish hospitals over the last two decades showed increasing clinical complexity and complications, with infections as the leading cause of admission and a modest rise in mortality despite a shorter LOS. These findings highlight the need to adapt healthcare models for this growing and vulnerable population.

**Supplementary Information:**

The online version contains supplementary material available at 10.1007/s41999-025-01362-1.

## Key summary points

**Aim:**

The aim of this study is to describe temporal trends in the clinical characteristics, principal diagnoses, and in-hospital outcomes of centenarian hospital admission in Spain (2004–2020).

**Findings:**

There was a notable shift toward infections as the leading cause of admission in centenarians along with significant increases in multimorbidity and in-hospital complications over the study period. Despite this, in-hospital mortality rose modestly, and hospital length of stay became shorter.

**Message:**

The growing clinical complexity in centenarian hospital admissions highlights the need for adapted care models, preventive strategies, and integrated interventions to avoid complications.

**Supplementary Information:**

The online version contains supplementary material available at 10.1007/s41999-025-01362-1.

## Introduction

One of the most illustrative phenomena of demographic change is the sustained growth of the centenarian population. Worldwide, the number of individuals aged 100 years or over is projected to rise from 441,000 in 2013 to over 20 million by 2100 [1]. This trend is mirrored in Spain, with an 89% increase between 2004 and 2020 [2], and projections estimating more than 100,000 centenarians by 2050 [3]. Similar demographic changes have been observed in countries such as Japan, France, and the Nordic region, although with considerable variability in centenarian prevalence, healthcare utilization patterns, and hospital outcomes [4, 5]. This age group has attracted increasing interest, not only as a model of exceptional longevity but also as a source of emerging challenges for healthcare systems, due to their increasing clinical complexity and diverse health trajectories [5].

Centenarians have traditionally been described as autonomous individuals with a low comorbidity burden and good functioning in whom the onset of serious diseases is postponed until the end of life [6, 7]. However, these findings mainly come from population-based studies conducted in the outpatient setting with heterogeneous methods and results, which limits their comparison and interpretation with the hospital setting [8, 9].

Hospitalization represents a critical point in these patients’ trajectory due to their high degree of frailty and risk of mortality [10]. In the last two decades, hospital admissions of centenarians have doubled in Spain and, although notable variability across regions and a slowdown in the rate of increase in recent years have been documented, the overall trend indicates that admissions continue to increase [2]. Indeed, this age group has had the greatest relative increase in admissions among those older than 85 years [11]. Despite this, there is scant evidence on the clinical profile, reasons for admission, and in-hospital progress of centenarians [12, 13].

In this context, characterizing the comorbidity and clinical outcomes of hospital admissions in these patients is essential to identify clinical patterns over time that allow for better healthcare planning and developing strategies aimed at reducing morbidity and mortality. Recent studies have shown that clinical variables derived from hospital records and administrative databases are a useful tool for the epidemiological analysis of this type of population [11, 14].

The aim of this study is to analyze the trends and changes in the clinical characteristics, principal diagnoses, and hospital outcomes of centenarian patients admitted to hospital in Spain over a period of more than 15 years.

## Methods

### Study population and data source

The method used in this study is consistent with that used in previous studies by this research group, which ensures analytical coherence and allows for comparing results [2, 12]. This research is based on a retrospective observational study conducted using a national registry that analyzed data on all unscheduled admissions of patients aged 100 years or older in the Spanish National Health System (SNS) from January 2004 to December 2020. The SNS is a publicly funded system that provides universal healthcare coverage across Spain's 17 autonomous communities and two autonomous cities. The SNS is decentralized, and each autonomous community has the capacity to manage its resources based on a common national healthcare framework [16].

The data were extracted from the Hospital Discharge Records-Minimum Basic Data Set (HDR-MBDS), a mandatory, standardized, and anonymized administrative registry that includes annual data on all hospital discharges from public and private hospitals. The HDR-MBDS contains summarized demographic and clinical data, including variables related to each hospital admission.

For every hospital admission, this work describes data including the year of admission, sex, age, admitting department, type of diagnosis-related group (DRG) (medical vs. surgical), principal diagnosis, secondary diagnoses (up to 20), procedures (up to 20), type of discharge (home, transfer to a residential facility, transfer to another hospital, or death), and length of hospital stay (LOS).

### Comorbidity and complications assessment

The principal diagnosis of each episode was used to define the main cause of hospitalization. It was coded according to the International Classification of Diseases, Clinical Modification (ICD-CM) (ICD-9-CM until 2015, and ICD-10-CM from 2016 onwards) and subsequently grouped into clinically relevant categories. Secondary diagnoses and procedures were analyzed using the same coding systems in order to identify chronic conditions, estimate comorbidity burden, and detect in-hospital complications. To avoid bias introduced by the transition from ICD-9 to ICD-10 and ensure longitudinal consistency, a harmonization process was implemented using custom software developed for this purpose. Diagnostic categories, including frailty-related diagnoses such as dementia, malnutrition, and urinary incontinence, were manually verified using correspondence tables from the WHO and the Spanish Ministry of Health to ensure semantic and clinical equivalence between coding systems.

A total of 32 chronic conditions were classified into 16 clinically homogeneous categories. Multimorbidity was defined as the presence of two or more chronic diseases in the same individual, consistent with validated criteria in population-based studies in this field [17]. Comorbidity burden was quantified using the Charlson Comorbidity Index (CCI), adapted for use with administrative databases. Severe comorbidity was defined as a CCI score greater than 2, which has been associated with an annual in-hospital mortality rate of more than 50% [18].

In-hospital complications were identified through secondary diagnostic coding. They included infections (respiratory tract infections (RTI), urinary tract infections (UTI), sepsis, and other infections), acute respiratory failure, acute kidney injury (AKI), hydroelectrolytic disorders, functional gastrointestinal disorders, anemia, malnutrition, delirium, pressure ulcers, and pharmacological complications.

To enhance the interpretability and clarity of the data presentation, variables deemed clinically irrelevant in the context and population under study were excluded from the final tables.

### Ethics approval and consent to participate

The HDR-MBDS is an anonymous, publicly accessible database intended for research use. It can be accessed by submitting a formal request to the Spanish Ministry of Health. Since all data are fully anonymized before being released, this study was exempt from the need for informed consent or ethics committee approval, in accordance with current Spanish regulations regarding the secondary use of health data for scientific research.

### Statistical analysis

This study examined trends in comorbidity burden, reasons for admission, and in-hospital outcomes according to sex among centenarian patients hospitalized between 2004 and 2020. Percent changes were calculated by subtracting the 2004 data from the 2020 data, dividing the remainder by the 2004 data, and expressing the result as a percentage. In addition, a log-linear joinpoint regression analysis was conducted to identify inflection points in the temporal trends of in-hospital outcomes, estimating the annual percent change (APC) with 95% confidence intervals. Up to three joinpoints were allowed in the models, with the optimal number selected based on the Bayesian information criterion (BIC).

A descriptive analysis was also performed for the clinical characteristics, principal diagnoses, and in-hospital complications of all centenarian admissions throughout the study period. Annual figures are detailed in the supplementary material. For clarity, results were grouped into three periods (2004–2010, 2011–2015, and 2016–2020). Quantitative variables were reported as means, standard deviations, and medians. Qualitative variables are reported as absolute and relative frequencies. The chi-square test for linear trend was used to assess associations between year, and qualitative variables and Spearman's correlation coefficient was used for quantitative variables. All analyses were two-tailed, and *p* values < 0.05 were considered statistically significant.

Statistical analyses were performed using SPSS^®^ software version 28 and Joinpoint Trend Analysis software version 4.9.1.0

## Results

### Changes in baseline clinical characteristics

Between 2004 and 2020, Spanish hospitals recorded 43,730 hospital discharges of people aged 100 or older, representing a 121.5% increase in their proportion relative to total hospital admissions. These corresponded to 31,753 patients, with readmissions accounting for 27.4% of the total number of cases. Table 1 summarizes the baseline characteristics over the three defined periods, while Supplementary Table 1 shows the year-by-year estimates and absolute differences.

**Table 1:** Changes in demographic and clinical characteristics of centenarians hospitalized in Spain, between 2004 and 2020

	2004–2010 *n* = 12,038	2011–2015 *n* = 13,508	2016–2020 *n* = 18,184	*p*
Age, years	101.6 ± 2.1 (101.0)	101.3 ± 1.9 (101.0)	101.3 ± 1.6 (101.0)	< 0.001
Sex, female	8545 (71.0)	10,270 (76.0)	14,176 (78.0)	< 0.001
Number of chronic diseases	2.2 ± 1.6 (2.0)	2.4 ± 1.8 (2.0)	3.0 ± 1.9 (3.0)	< 0.001
Multimorbidity	7387 (61.4)	8597 (63.6)	13,937 (76.6)	< 0.001
CCI	1.3 ± 1.4 (1.0)	1.5 ± 1.6 (1.0)	1.9 ± 1.7 (2.0)	< 0.001
Severe comorbidity, CCI ≥ 3	2124 (17.6)	3003 (22.2)	5791 (31.8)	< 0.001
Place of residence, nursing home	1424 (11.8)	1373 (10.2)	2146 (11.8)	0.615
Chronic conditions				
Hypertension	4585 (38.1)	5223 (38.7)	7008 (38.5)	0.475
CHF	3422 (28.4)	3774 (27.9)	5651 (31.1)	< 0.001
AF	2582 (21.4)	3040 (22.5)	4511 (24.8)	< 0.001
Anemia	2429 (20.2)	2749 (20.4)	3517 (19.3)	0.050
CKD	2052 (17.0)	2474 (18.3)	3757 (20.7)	< 0.001
Dementia	1988 (16.5)	2273 (16.8)	3624 (19.9)	< 0.001
Diabetes	1568 (13.0)	1681 (12.4)	2391 (13.1)	0.597
CVD	1378 (11.4)	1663 (12.3)	2772 (15.2)	< 0.001
COPD	1268 (10.5)	1372 (10.2)	1953 (10.7)	0.452
CAD	1142 (9.5)	1403 (10.4)	2029 (11.2)	< 0.001
Hearing loss	1134 (9.4)	1224 (9.1)	1587 (8.7)	0.039
Visual loss	967 (8.0)	1199 (8.9)	1552 (8.5)	0.191
Dyslipidemia	952 (7.9)	1294 (9.6)	1852 (10.2)	< 0.001
Other arrhythmias	947 (7.9)	1080 (8.0)	1418 (7.8)	0.775
Active malignant neoplasm	722 (6.0)	996 (7.4)	1128 (6.2)	0.887
Thyroid disease	619 (5.1)	728 (5.4)	1029 (5.7)	0.050
Biliopancreatic disease	587 (4.9)	1076 (8.0)	1097 (6.0)	0.003
Pressure ulcers	565 (4.7)	841 (6.2)	1279 (7.0)	< 0.001
Urinary incontinence	514 (4.3)	781 (5.8)	1104 (6.1)	< 0.001

Continuous variables are expressed as mean ± standard deviation (median) and categorical variables as number (percentage). Multimorbidity = ≥ 2 chronic diseases

*CCI* Charlson Comorbidity index, *CHF* congestive heart failure, *AF* atrial fibrillation, *CKD* chronic kidney disease, *CVD* cerebrovascular disease, *COPD* chronic obstructive pulmonary disease, *CAD* coronary artery disease

**Fig. 1:**
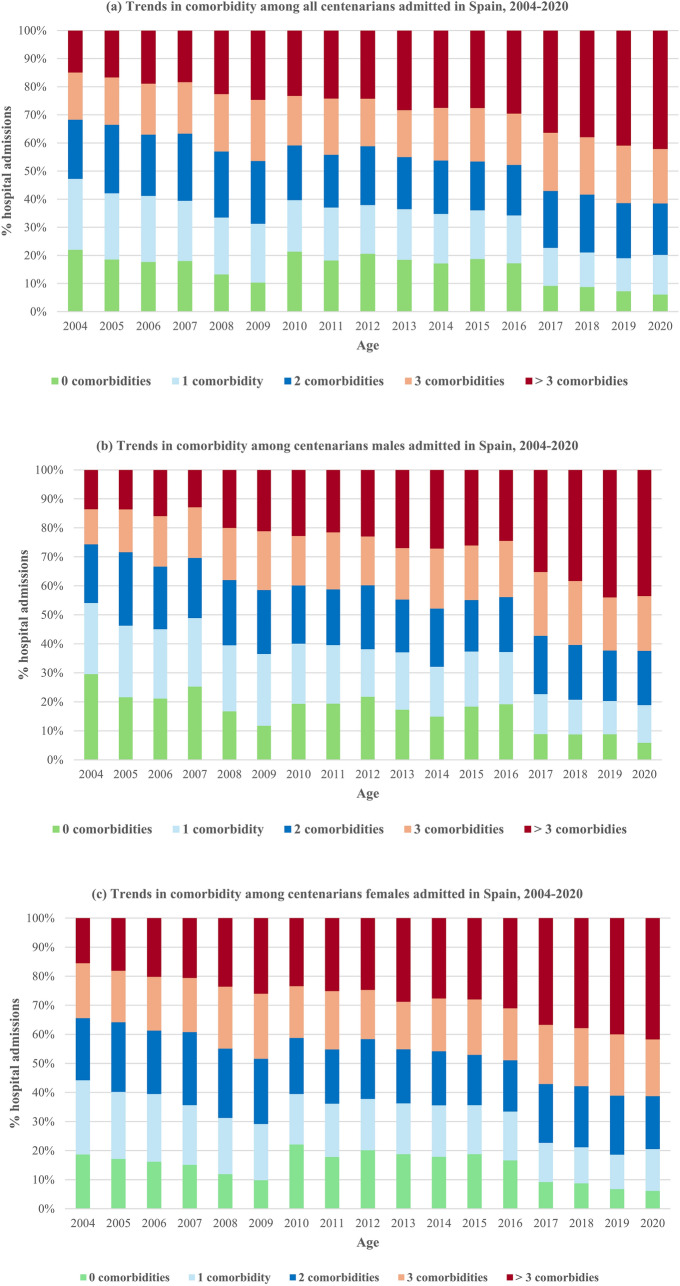
Trends in comorbidity among all hospital admissions in centenarians in Spain, 2004–2020. **a** Trends in comorbidity among all centenarian patients, **b** trends in comorbidity among centenarian males, **c** trends in comorbidity among centenarian females

A consistent trend toward increased clinical complexity was observed. The mean CCI increased from 1.3 ± 1.4 in 2004–2010 to 1.9 ± 1.7 in 2016–2020 (*p* < 0.001), while the prevalence of severe comorbidity nearly doubled (from 17.6 to 31.8%, *p* < 0.001). Similarly, the mean number of chronic conditions increased from 2.2 ± 1.6 to 3.0 ± 1.9 (*p* < 0.001), while the prevalence of multimorbidity increased from 61.4 to 76.6% (*p* < 0.001). This trend is particularly evident in the proportion of centenarians hospitalized without any chronic condition, which fell from 22 to 6.1%, in contrast to those with 3 or more conditions, which rose from 16.7 to 42.2% (*p* < 0.001), in both males and females. These trends were consistently observed in both sexes throughout the study period **(**Fig. 1), although there were certain differences between them. While men had higher CCI scores and a higher prevalence of severe comorbidity in all periods, the relative increase over time was more pronounced in women. Conversely, women had a higher number of chronic diseases and multimorbidity, but the increase over the study period was more marked among men (Supplementary Table 2).

A parallel trend was observed in baseline comorbidities across the study periods, especially for those related to cardiovascular-kidney-metabolic syndrome (dyslipidemia, coronary artery disease (CAD), chronic heart failure (CHF), atrial fibrillation (AF), cerebrovascular disease (CVD), and chronic kidney diseases (CKD)), biliopancreatic disease, and geriatric syndromes and frailty (dementia, urinary incontinence, hearing loss, and pressure ulcers). The most pronounced relative increases from 2004 to 2020 were observed in dyslipidemia (2.3–19.5%), thyroid diseases (2.2–9.8%), urinary incontinence (2.3–8.5%), CKD (8.2–29.3%), pressure ulcers (4.1–7.9%), and dementia (12.9–23.7%). These trends were similar among men and women (Supplementary Table 2).

The predominance of females also followed an upward trend, increasing from 71% of the total centenarian population in 2004–2010 to 78% in 2016–2020 (*p* < 0.001). The proportion of patients admitted from nursing homes remained stable.

### Changes in principal diagnosis

A significant trend was noted toward a higher proportion of medical admissions, increasing from 82.2% in 2004–2010 to 86.1% in 2016–2020 (*p* < 0.001), with a 14.1% relative absolute increase from 2004 to 2020. On the other hand, surgical admissions declined by 45% (*p* < 0.001) (Table 2, Supplementary Table 3).

**Table 2:** Changes in principal diagnosis of centenarians hospitalized in Spain, between 2004 and 2020

	2004–2010 *n* = 12,038	2011–2015 *n* = 13,508	2016–2020 *n* = 18,184	*p*
DRG type, medical	9896 (82.2)	11,642 (86.2)	15,660 (86.1)	< 0.001
Principal diagnosis				
Infections, total	3297 (27.4)	4337 (32.1)	7020 (38.6)	< 0.001
RTI	2214 (18.4)	2869 (21.2)	4741 (26.1)*	< 0.001
Pneumonia	1534 (12.7)	2065 (15.3)	2610 (14.3)	< 0.001
Acute bronchitis	441 (3.7)	560 (4.1)	1475 (8.1)	< 0.001
COPD exacerbation	235 (2.0)	226 (1.7)	301 (1.7)	0.067
UTI	509 (4.2)	822 (6.1)	1442 (7.9)	< 0.001
Intraabdominal infection	283 (2.4)	310 (2.3)	495 (2.7)	0.027
Sepsis, without defined focus	124 (1.0)	234 (1.7)	119 (0.7)	< 0.001
HF	1376 (11.4)	1756 (13.0)	2240 (12.3)	0.053
Hip fracture	1249 (10.4)	1401 (10.4)	1612 (8.9)	< 0.001
Acute CVD	675 (5.6)	732 (5.4)	839 (4.6)	< 0.001
Acute Respiratory Failure	488 (4.1)	644 (4.8)	643 (3.5)	0.005
Active malignant neoplasm	383 (3.2)	322 (2.3)	413 (2.3)	< 0.001
Fracture, other than a hip fracture	367 (3.0)	343 (2.5)	472 (2.6)	0.028
Acute Ischemic Heart Disease	298 (2.5)	265 (2.0)	267 (1.5)	< 0.001
Gastrointestinal bleeding	270 (2.2)	294 (2.2)	367 (2.0)	0.171
Acute Arterial Occlusion	254 (2.1)	269 (2.0)	258 (1.4)	< 0.001
Arrhythmias	248 (2.0)	290 (2.1)	296 (1.6)	0.013
Biliopancreatic disease	193 (1.6)	245 (1.8)	226 (1.2)	0.004
Acute gastroenteritis and enterocolitis	188 (1.6)	185 (1.4)	239 (1.3)	0.082
Hydroelectrolytic disorders	165 (1.4)	189 (1.4)	220 (1.2)	0.188
AKI	139 (1.2)	221 (1.6)	636 (1.5)	0.020
Anemia	117 (1.0)	163 (1.2)	207 (1.1)	0.231

Continuous variables are expressed as mean ± standard deviation and categorical variables as number (percentage)

*DRG* diagnosis-related group, *RTI* respiratory tract infection, *COPD* chronic obstructive pulmonary disease, *UTI* urinary tract infection, *HF* heart failure, *CVD* cerebrovascular disease, *AKI* acute kidney injury

*The number of hospital admissions for SARS-CoV-2 in 2020 was 211 (1.2% of total admissions between 2016 and 2020), and the percentage of total RTI between 2016 and 2020 excluding SARS-CoV-2 hospital admissions was 24%

Regarding the principal diagnoses, infections, heart failure (HF), and hip fracture became the leading causes of admission over the three periods. However, of these, only infections showed an upward trend (*p* < 0.001), mainly due to RTI (from 18.4% in 2004–2010 to 26.1% in 2016–2020) and UTI (4.2–7.9%). In contrast, a declining trend was observed in hip fracture (from 10.4 to 8.9%, *p* < 0.001) and figures remained stable for HF. Although 2020 includes the first wave of the COVID-19 pandemic, admissions with confirmed SARS-CoV-2 infection accounted for only 1.2% of total cases and did not substantially alter the overall trends.

In addition, there was also a trend toward a decline in admissions for acute CVD (5.6–4.6%, *p* < 0.001), acute respiratory failure (4.1–3.5%, *p* = 0.005), other fractures (3–2.6%, *p* = 0.028), and active malignant neoplasm (3.2–2.3%, *p* < 0.001).

**Fig. 2:**
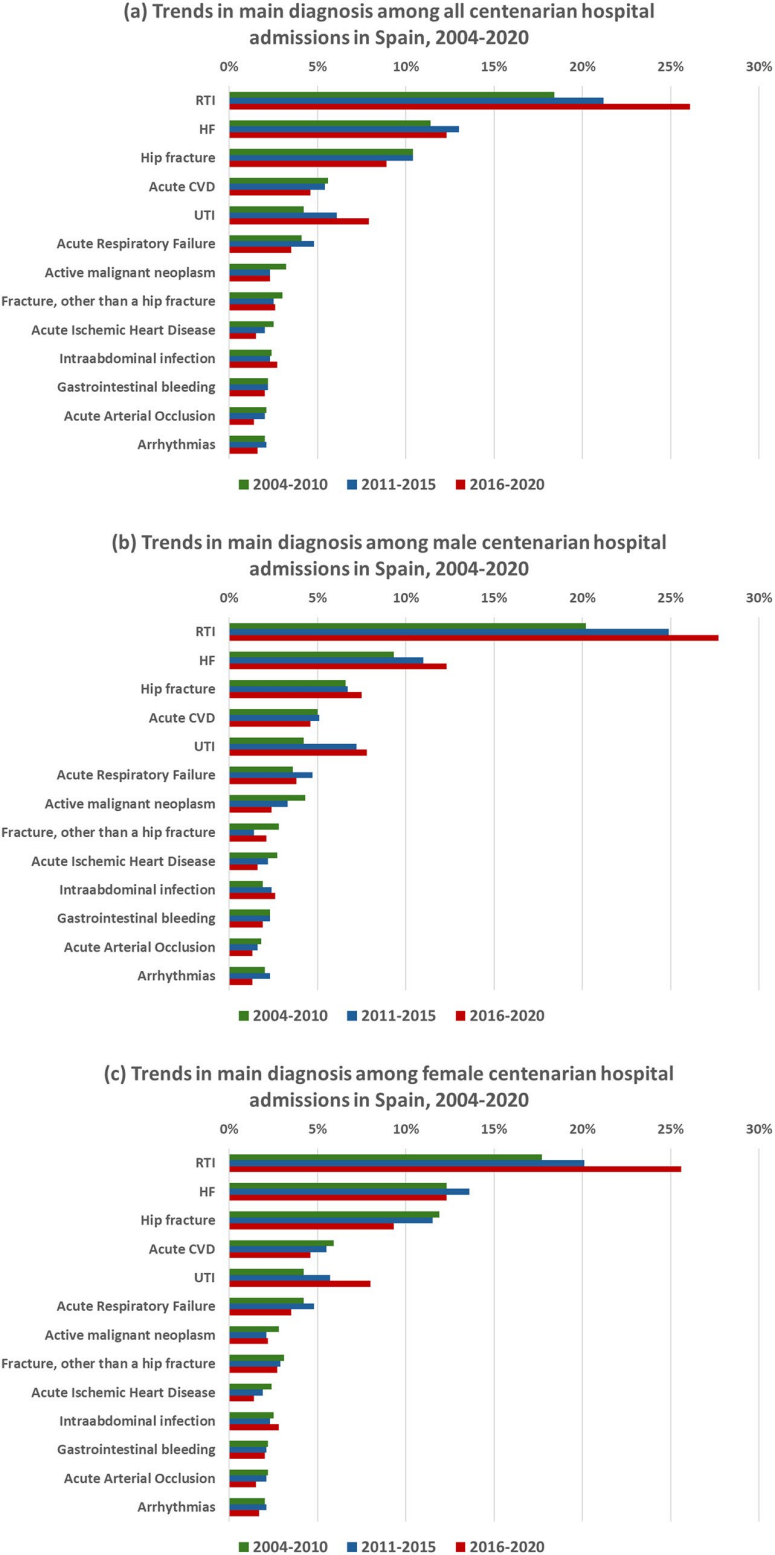
Sex-based trends in principal diagnosis of centenarian hospital admissions in Spain (2004–2020). **a** Trends in main diagnosis among all centenarians, **b** trends in main diagnosis among male centenarians, **c** trends in main diagnosis among female centenarians. *RTI* respiratory tract infection, *HF* heart failure, *CVD* cerebrovascular disease, *UTI* urinary tract infection

These trends were generally similar between men and women, though a notable increase in admissions due to HF and hip fracture was observed in men, whereas these diagnoses remained stable or declined, respectively, among women. (Fig. 2, Supplementary Table 4).

### Trends in in-hospital outcomes

**Fig. 3:**
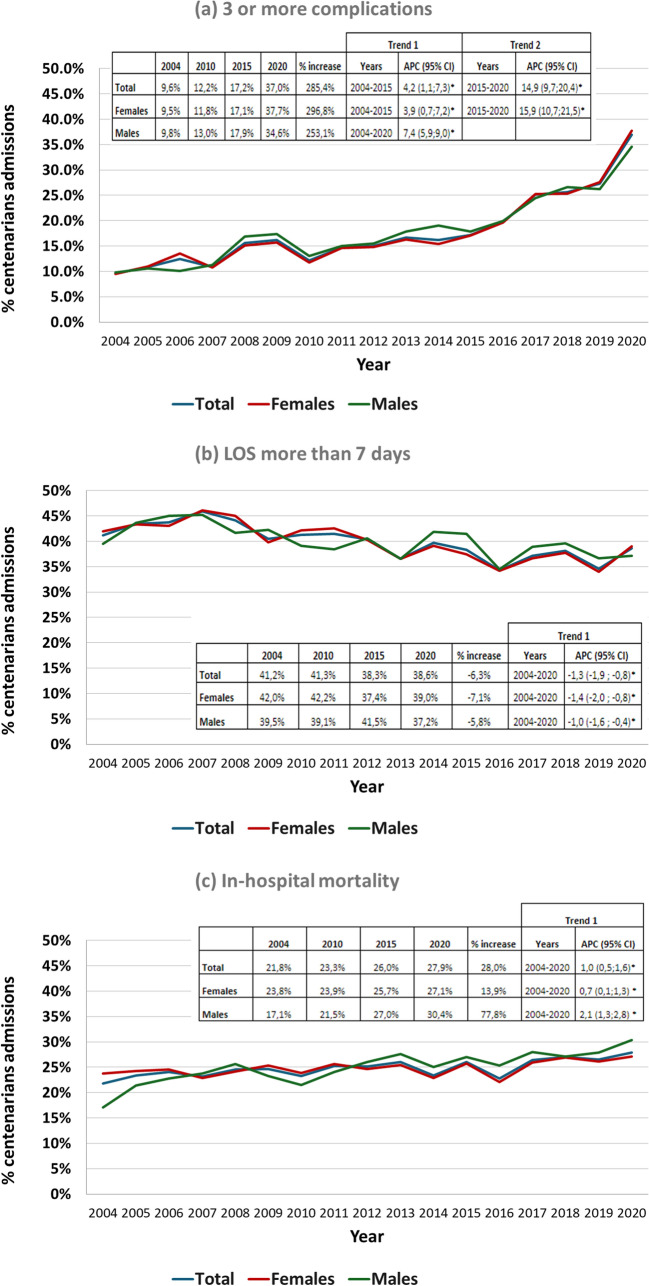
Sex-based trends in in-hospital outcomes of centenarian hospital admissions in Spain (2004–2020). **a** % patients with 3 or more in-hospital complications, **b** % patients with more than a week of length of stay, **c** % in-hospital mortality. *APC* annual percent change, *CI* confidence interval, *LOS* length of hospital stay

A clear upward trend in in-hospital complications was observed across the study periods. This trend was more pronounced in the proportion of patients with 3 or more complications (from 12.8% in 2004–2010 to 26.8% in 2016–2020), while those without complications declined (46.2–31.4%, *p* < 0.001) (Table 3) between 2015 and 2020 (APC = 14.9%; 95% CI 9.7%; 20.4%), mainly among women (APC = 15.9%; 95% CI 10.7%; 21.5%) (Fig. 3). All categories of complications showed significant upward trends (*p* < 0.001), with the most marked increases observed in malnutrition (2.6% in 2004 to 13.4% in 2020), sepsis (1.0–4.4%), and AKI (5.0–20.5%) (Supplementary Table 5).

**Table 3:** Changes in outcomes related to hospital admissions of centenarians in Spain, between 2004 and 2020

	2004–2010 *n* = 12,038	2011–2015 *n* = 13,508	2016–2020 *n* = 18,184	*p*
In-hospital complications				
Acute respiratory failure	2114 (17.6)	2467 (18.3)	4347 (23.9)	< 0.001
Anemia	1844 (15.3)	2306 (17.1)	4058 (22.3)	< 0.001
Hydroelectrolytic disorders	1210 (10.1)	1463 (10.8)	2911 (16)	< 0.001
Other infections	893 (7.4)	1136 (8.4)	2525 (13.9)	< 0.001
AKI	865 (7.2)	1606 (11.9)	3292 (18.1)	< 0.001
UTI	860 (7.1)	986 (7.3)	1904 (10.5)	< 0.001
RTI	740 (6.1)	531 (3.9)	1176 (6.5)	0.019
Delirium	679 (5.6)	761 (5.6)	1520 (8.4)	< 0.001
Pressure ulcers	577 (4.8)	720 (5.3)	1148 (6.3)	< 0.001
Functional gastrointestinal disorders	522 (4.3)	829 (6.1)	1470 (8.1)	< 0.001
Malnutrition	514 (4.3)	701 (5.2)	2009 (11.0)	< 0.001
Sepsis	240 (2.0)	371 (2.7)	814 (4.5)	< 0.001
*No. in-hospital complications*				
None	5562 (46.2)	6202 (45.9)	5708 (31.4)	< 0.001
1	3409 (28.3)	3275 (24.2)	4522 (24.9)	
2	1531 (12.7)	1861 (13.8)	3072 (16.9)	
≥ 3	1536 (12.8)	2170 (16.1)	4882 (26.8)	
LOS, days	8.7 ± 10.5 (6.0)	7.7 ± 7.7 (6.0)	7.3 ± 7.3 (6.0)	< 0.001
Survivors	9.0 ± 10.7 (7.0)	7.9 ± 7.8 (6.0)	7.5 ± 7.2 (6.0)	
Non-survivors	7.8 ± 9.6 (5.0)	7.0 ± 7.4 (5.0)	6.7 ± 7.5 (5.0)	
Place of discharge, nursing home	256 (2.1)	481 (3.6)	865 (4.8)	< 0.001

Continuous variables are expressed as mean ± standard deviation and categorical variables as number (percentage)

*AKI* acute kidney injury, *UTI* urinary tract infection, *RTI* respiratory tract infection, *LOS* length of hospital stay

The average LOS showed a consistent downward trend, decreasing from 8.7 ± 10.5 days in 2004–2010 to 7.3 ± 7.3 days in 2016–2020 (*p* < 0.001), with an overall annual decrease in the percentage of patients with LOS longer than seven days of 1.3% (APC = − 1.3%; 95% CI − 1.9%;− 0. 8%); the changes were similar in both sexes (Table 3; Fig. 3, Supplementary Table 6).

A total of 10,989 (25.1%) centenarians died during their hospital stay. Overall, in-hospital mortality showed a modest but upward trend throughout the study period (APC = 1. 0%; 95% CI 0.5%; 1.6%) that was more pronounced in men (APC = 2.1%; 95% CI 1.3%; 2.8%) than in women (APC = 0.7%; 95% CI 0.1%; 1.3%) (Fig. 3).

Finally, the proportion of discharges to nursing homes more than doubled, from 2.1% in 2004–2010 to 4.8% in 2016–2020 (*p* < 0.001).

## Discussion

This study provides a comprehensive analysis of temporal trends in the clinical profile, reasons for hospital admission, and in-hospital outcomes of centenarians in Spain. It is the most extensive hospital series of this nature published to date. Among the most relevant findings is a sustained increase in clinical complexity, characterized by a higher prevalence of multimorbidity, severe comorbidity, and in-hospital complications as well as the predominance of infections as the main reason for admission. There was also a modest but significant increase in mortality. On the other hand, LOS declined significantly, and discharges to residential facilities doubled, suggesting changes in acute care models and social–healthcare coordination.

The clinical characteristics of centenarian patients are very heterogeneous [5]. Several phenotypes have been proposed to characterize their profile based on the compression of morbidity and the time of onset of more severe conditions [6, 19, 20]. However, methodological variability, particularly in the definitions and in the type and number of chronic conditions considered [9, 13], limits comorbidity stratification and cross-cohort comparability. Our data reveal a sustained increase in both multimorbidity and severe comorbidity over time, suggesting a transition from the traditional “healthy aging” phenotype to a more complex and frail clinical profile at hospital admission.

Notably, the prevalence of dementia increased steadily throughout the study period, reaching nearly 24% in 2020. Although likely underestimated due to limitations in stratified data capture from administrative databases, its clinical relevance is well established. Dementia is strongly associated with in-hospital complications (e.g., delirium, malnutrition, pressure ulcers), care dependency, and institutionalization [3]. In contrast, the increase observed in certain conditions (such as dyslipidemia, CKD, thyroid disease, or other geriatric syndromes) could be influenced by improvements in the quality of diagnostic coding practices over time [2].

This transformation is in line with contemporary European series [6, 7, 20] and probably reflects two complementary dimensions of extreme aging. First, the differential resilience of centenarians who, while not escaping physiological decline and age-related diseases, have the ability to mitigate the speed of their deleterious effects, live longer with multimorbidity, and postpone the onset of serious conditions to the later years of life [7, 21–23]. Second, a growing burden of frailty not adequately captured by traditional comorbidity indices [24, 25]. Frailty, as defined by the cumulative deficit model, is characterized by an increased vulnerability to stressors and reduced physiological reserve, and has been associated with greater cognitive, physical and social vulnerability, as well as adverse outcomes and early institutionalization in this age group [7, 10, 26].

In this cohort, admissions of centenarians are mainly due to medical DRG and infections (mainly respiratory), which have become established as the main cause of admission. On the contrary, there was a reduction in admissions for surgical DRG and severe acute diseases such as proximal hip fracture (PHF), acute CVD, or acute malignant neoplasm. Admissions for HF remained stable despite the increase in cardiovascular comorbidity. This pattern may be due to multiple factors: the growing influence of immunosenescence and functional abnormalities associated with extreme aging [8, 10, 22] a lower incidence of rapidly lethal diseases in this population selected for biological resilience; and a more conservative clinical attitude toward severe diseases, which prioritizes palliative care or home management in terminal phases [7, 27, 28]. In addition, recent improvements in community-based care models for chronic conditions in Spain may be channeling potential avoidable hospital admissions through alternative pathways rather than conventional hospital care [2]. Moreover, the etiological distribution of centenarian hospital admissions in our study coincides with that reported in other national, European, and American series [24, 25, 29–31].

Although in-hospital complications in centenarians are frequent and those who develop them have worse health outcomes, very few studies evaluate them at the in-hospital level and most are in patients admitted for PHF [32, 33]. The sustained increase in the frequency and number of all hospital complications (including delirium, malnutrition, nosocomial infections, or renal and metabolic disorders) in our study reinforces the vulnerability of centenarians to the stress of hospitalization [13, 31, 34] especially women [8], and the possibility that cascade iatrogenesis may be triggered with the onset of multiple complications [35]. This phenomenon could be attributed to rapid physical deterioration during admission, baseline frailty, or better early detection of these events through proactive surveillance strategies [15, 24].

The in-hospital mortality observed in various series, which is stable and situated between 16 and 31%, reflects this population’s high in-hospital mortality rate, especially due to acute respiratory infectious diseases (the main cause of death) and particularly in males [36–38], as shown longitudinally in this work. These differences according to sex together with those observed in the pattern of morbidity and reasons for admission reinforce the hypothesis of divergent aging trajectories [22, 37]. Women, despite having a greater biological reserve, present with geriatric syndromes more frequently and have higher levels of disability, while men show a more severe comorbidity burden, clinical episodes of greater severity, and a worse vital prognosis [7, 11, 30, 31].

The significant reduction in LOS, observed despite increase clinical complexity, can be explained by a combination of factors reflecting both changes in hospital clinical care and improvements in healthcare organization that have facilitated early discharge. These include the reorganization of acute care pathways, which is being driven by growing pressure to optimize bed occupancy and streamline emergency services in order to reduce healthcare costs [13, 30, 39]; improvements in perioperative care, such as early mobilization protocols and the adoption of comanagement models [33, 40]; the implementation of more inclusive therapeutic approaches that have overcome previous age-based limitations [13]; and a progressive shift toward goal-oriented clinical decisions that prioritize quality of life over the futile prolongation of hospitalization for patients with poor prognosis [27]. Lastly, the uneven but evolving development of community-based care models in Spain, especially in rural regions with stronger family support systems, may also have contribute to shorter LOS in end-of-life contexts [20, 24, 27].

Despite having doubled over the study period, the proportion of centenarians admitted to or discharged from residential care facilities in Spain remained notably low (2.1–4.8%) compared to figures reported in other European and North American contexts (45–90%) [4, 29]. This disparity likely reflects structural and cultural features of the Spanish long-term care system, including a lower density of nursing home beds and the use of such facilities primarily as short-term transitional resources after hospitalization [2, 24]. Moreover, Spain maintains a strong reliance on informal caregiving networks, especially in rural regions, where family-based support often substitutes for institutional care [3, 42]. These characteristics differ from countries such as Sweden or Switzerland, with lower hospitalization rates among centenarians, particularly in the last year of life, may reflect greater integration of residential care into the continuum of health services [5, 7, 10]. In contrast, rates of hospitalization exceed 50% in the United States and the United Kingdom, often for conditions considered potentially avoidable [4, 30]. In Japan, advanced age is associated with both lower end-of-life hospital use and reduced healthcare expenditures [41]. These international contrasts underscore the influence of health system configuration, long-term care infrastructure, and cultural preferences on hospital use patterns in centenarian populations [5, 20, 36].

These changes observed in the clinical profile, reasons for admission, and in-hospital progress of centenarians reflect not only individual biological and clinical dynamics but also a broader transformation in the way healthcare systems respond to extreme aging. In this sense, it is key to analyze the organizational and public health implications that emerge from these findings. The increasing presence of centenarians in the hospital setting poses new challenges for European healthcare systems. This population requires integrated, person-centered, realistic models of care capable of responding to cases of high clinical frailty and functional dependence [43, 44]. Furthermore, these results support the need to reinforce multidisciplinary preventive measures to address complications [35], social–healthcare coordination between levels of care, and the exploration of new quality indicators adjusted to this age profile.

The main advantages of this work are that it is based on a population cohort studies over a long period of time and the large sample size. It is the longest series of centenarians described in literature. These characteristics allow the study population to be represented in a highly accurate manner and provide remarkable statistical power for the analysis of demographic and clinical variables. This is especially relevant for a growing but still small cohort, such as centenarians. In addition, the data on hospital discharges are drawn from a primary, official administrative source of clinical information that has been standardized. It represents practically all Spanish hospitals and has been proven useful in biomedical research [11, 14].

Nonetheless, several limitations must be considered. First, the quality of the records in administrative databases depends on clinical coding, which may induce under-recording or errors in the classification of diagnoses, especially in populations with atypical characteristics and presentations such as centenarians [12]. Secondly, this database lacks clinical information that is significant for a comprehensive assessment, such as functional status, treatments administered, progress after discharge, or cause of death. This limits the ability to evaluate the adequacy of clinical management and the impact of hospitalization on quality of life. Finally, the retrospective, descriptive nature of this study limits the possibility of establishing causal relationships between clinical variables and the trends identified.

To further the understanding of these findings, prospective research that incorporates longitudinal follow-up and an assessment of prognostic factors is required. This approach would allow not only for better elucidating the underlying mechanisms but also for designing effective strategies to prevent hospital complications in a centenarian population that is growing in number and clinical complexity.

## Conclusion

The clinical profile of centenarians admitted to Spanish hospitals between 2004 and 2020 became increasingly complex, with a higher prevalence of multimorbidity and severe comorbidity. Infections emerged as the main cause of admission, and in-hospital complications increased significantly. Despite this, mortality increased only modestly, and the LOS decreased. These findings underscore the need to adapt healthcare models for this growing and vulnerable population. Future prospective studies are needed to evaluate prognostic factors and help design effective strategies to avoid complications.

## Supplementary Information

Below is the link to the electronic supplementary material.

Supplementary file1 (PDF 662 KB)

## References

[1] Afonso RM, Ribeiro O, Vaz Patto M, Loureiro M, Loureiro MJ, Castelo-Branco M, Patrício S, Alvarinhas S, Tomáz T, Rocha C, Jerónimo AM, Gouveia F, Amaral AP (2018) Reaching 100 in the countryside: health profile and living circumstances of Portuguese centenarians from the Beira Interior Region. Curr Gerontol Geriatr Res 2018:8450468. 10.1155/2018/8450468 30008746 10.1155/2018/8450468 PMC6020501

[2] Piñeiro-Fernández JC, Rabuñal-Rey R, Maseda A, Romay-Lema E, Suárez-Gil R, Pértega-Díaz S (2024) Demographic transition and hospital admissions in Spanish centenarians, 2004-2020: geographical variations and sex-related differences. Arch Gerontol Geriatr 117:105276. 10.1016/j.archger.2023.105276 37984196 10.1016/j.archger.2023.105276

[3] Vega-Alonso T, Lozano-Alonso J, Estévez-Iglesias L, Ordax-Díez A, Arrieta-Antón E, Díaz-Rodríguez Á, Yañez-Ortega JL, Santos-Lozano A, Nuñez-Torres R, Perez-Caro M, Pita G, Pinto-Labajo R, Alonso Ramos MJ, Álamo-Sanz R, García-Montero AC, Gonzalez-Neira A (2024) Health and wellbeing status of the long-lived individuals of the Spanish LONGECYL cross-sectional study. Arch Public Health 82(1):77. 10.1186/s13690-024-01305-5 38769585 10.1186/s13690-024-01305-5 PMC11103821

[4] Dupraz J, Andersen-Ranberg K, Fors S, Herr M, Herrmann FR, Wakui T, Jeune B, Robine JM, Saito Y, Santos-Eggimann B, 5-COOP group (2020) Use of healthcare services and assistive devices among centenarians: results of the cross-sectional, international 5-COOP study. BMJ Open 10(3):e034296. 10.1136/bmjopen-2019-034296 32209627 10.1136/bmjopen-2019-034296 PMC7202712

[5] Jopp DS, Boerner K, Ribeiro O, Rott C (2016) Life at age 100: an international research agenda for centenarian studies. J Aging Soc Policy 28(3):133–147. 10.1080/08959420.2016.1161693 26984376 10.1080/08959420.2016.1161693

[6] Andersen-Ranberg K, Schroll M, Jeune B (2001) Healthy centenarians do not exist, but autonomous centenarians do: a population-based study of morbidity among Danish centenarians. J Am Geriatr Soc 49(7):900–908. 10.1046/j.1532-5415.2001.49180.x 11527481 10.1046/j.1532-5415.2001.49180.x

[7] Vetrano DL, Grande G, Marengoni A, Calderón-Larrañaga A, Rizzuto D (2021) Health trajectories in Swedish centenarians. J Gerontol A Biol Sci Med Sci 76(1):157–163. 10.1093/gerona/glaa152 32569349 10.1093/gerona/glaa152 PMC7756707

[8] Hazra NC, Dregan A, Jackson S, Gulliford MC (2015) Differences in health at age 100 according to sex: population-based cohort study of centenarians using electronic health records. J Am Geriatr Soc 63(7):1331–1337. 10.1111/jgs.13484 26096699 10.1111/jgs.13484 PMC4745036

[9] Poon LW, Jazwinski M, Green RC, Woodard JL, Martin P, Rodgers WL, Johnson MA, Hausman D, Arnold J, Davey A, Batzer MA, Markesbery WR, Gearing M, Siegler IC, Reynolds S, Dai J (2007) Methodological considerations in studying centenarians: lessons learned from the Georgia Centenarian Studies. Annu Rev Gerontol Geriatr 27(1):231–264 21852888 PMC3156654

[10] Herr M, Jeune B, Fors S, Andersen-Ranberg K, Ankri J, Arai Y, Cubaynes S, Santos-Eggimann B, Zekry D, Parker M, Saito Y, Herrmann F, Robine JM, 5-COOP group (2018) Frailty and associated factors among centenarians in the 5-COOP countries. Gerontology 64(6):521–531. 10.1159/000489955 30032145 10.1159/000489955

[11] Palacios-Fernandez S, Salcedo M, Gonzalez-Alcaide G, Ramos-Rincon JM (2021) Time trends in hospital discharges in patients aged 85 years and older in Spain: data from the Spanish National Discharge Database (2000-2015). BMC Geriatr 21(1):371. 10.1186/s12877-021-02335-2 34134638 10.1186/s12877-021-02335-2 PMC8207637

[12] Brandão D, Ribeiro O, Freitas A, Paúl C (2017) Hospital admissions by the oldest old: past trends in one of the most ageing countries in the world. Geriatr Gerontol Int 17(11):2255–2265. 10.1111/ggi.13006 28276619 10.1111/ggi.13006

[13] Engberg H, Oksuzyan A, Jeune B, Vaupel JW, Christensen K (2009) Centenarians–a useful model for healthy aging? A 29-year follow-up of hospitalizations among 40,000 Danes born in 1905. Aging Cell 8(3):270–276. 10.1111/j.1474-9726.2009.00474.x 19627266 10.1111/j.1474-9726.2009.00474.x PMC2774420

[14] Caballero-Segura FJ, Lopez-de-Andres A, Jimenez-Garcia R, de Miguel-Yanes JM, Hernández-Barrera V, Carabantes-Alarcon D, Zamorano-Leon JJ, de Miguel-Díez J (2022) Trends in asthma hospitalizations among adults in Spain: analysis of hospital discharge data from 2011 to 2020. Respir Med 204:107009. 10.1016/j.rmed.2022.107009 36265419 10.1016/j.rmed.2022.107009

[15] Piñeiro-Fernández JC, Rabuñal-Rey R, Romay-Lema E, Pedrosa-Fraga C, Rubal-Bran D, Suárez-Gil R, Marchán-López Á, Pértega-Díaz S (2025) Epidemiology and trends of hip fracture in centenarians: changes in clinical profile and in-hospital outcomes from a nationwide register study in Spain across 2004–2020. Aging Clin Exp Res 37(1):84. 10.1007/s40520-025-02994-w 40074889 10.1007/s40520-025-02994-w PMC11903550

[16] Yetano-Laguna J, Laraudogoitia-Zaldumbide E (2007) Documentación clínica. Aspectos legales y fuente de información para las bases de datos hospitalarias. Rev Esp Cardiol Supl 7(3):2C-11C. 10.1016/S1131-3587(07)75244-5

[17] Matesanz-Fernández M, Seoane-Pillado T, Iñiguez-Vázquez I, Suárez-Gil R, Pértega-Díaz S, Casariego-Vales E (2022) Description of multimorbidity clusters of admitted patients in medical departments of a general hospital. Postgrad Med J 98(1158):294–299. 10.1136/postgradmedj-2020-139361 33547138 10.1136/postgradmedj-2020-139361

[18] Charlson ME, Carrozzino D, Guidi J, Patierno C (2022) Charlson comorbidity index: a critical review of clinimetric properties. Psychother Psychosom 91(1):8–35. 10.1159/000521288 34991091 10.1159/000521288

[19] Brandão D, Ribeiro O, Afonso RM, Paúl C (2019) Regional differences in morbidity profiles and health care use in the oldest old: findings from two Centenarian Studies in Portugal. Arch Gerontol Geriatr 82:139–146. 10.1016/j.archger.2019.02.009 30797992 10.1016/j.archger.2019.02.009

[20] Clerencia-Sierra M, Ioakeim-Skoufa I, Poblador-Plou B, González-Rubio F, Aza-Pascual-Salcedo M, Gimeno-Miguel MMA, Prados-Torres A (2020) Do centenarians die healthier than younger elders? A comparative epidemiological study in Spain. J Clin Med 9(5):1563. 10.3390/jcm9051563 32455809 10.3390/jcm9051563 PMC7291259

[21] Rasmussen SH, Andersen-Ranberg K. Health in centenarians. In: Pachana N, editor. Encyclopedia of Geropsychology. Singapore: Springer; 2016. 10.1007/978-981-287-080-3_78-1.

[22] Borras C, Ingles M, Mas-Bargues C, Dromant M, Sanz-Ros J, Román-Domínguez A, Gimeno-Mallench L, Gambini J, Viña J (2020) Centenarians: an excellent example of resilience for successful ageing. Mech Ageing Dev 186:111199. 10.1016/j.mad.2019.111199 31899226 10.1016/j.mad.2019.111199

[23] Gellert P, von Berenberg P, Zahn T, Neuwirth J, Kuhlmey A, Dräger D (2019) Multimorbidity profiles in German centenarians: a latent class analysis of health insurance data. J Aging Health 31(4):580–594. 10.1177/0898264317737894 29254430 10.1177/0898264317737894

[24] Sáez-Nieto C, Ly-Yang F, Pérez-Rodríguez P, Alarcón T, López-Arrieta J, González-Montalvo JI (2019) Impact of hospital admission on centenarians admitted due to an acute illness. A description of a series of 165 cases and comparison with the literature. Rev Esp Geriatr Gerontol 54(6):315–320. 10.1016/j.regg.2019.04.005 31301820 10.1016/j.regg.2019.04.005

[25] Tello Rodríguez T, Varela Pinedo LF, Ortiz Saavedra PJ, Guevara Linares X (2017) Health status of centenarians in a general hospital in Lima-Perú. Rev Esp Geriatr Gerontol 52(1):54–55. 10.1016/j.regg.2016.04.003 27264616 10.1016/j.regg.2016.04.003

[26] Rockwood K, Song X, MacKnight C, Bergman H, Hogan DB, McDowell I, Mitnitski A (2005) A global clinical measure of fitness and frailty in elderly people. CMAJ 173(5):489–495. 10.1503/cmaj.050051 16129869 10.1503/cmaj.050051 PMC1188185

[27] Chen YC, Hu HY, Fan HY, Kao WS, Chen HY, Huang SJ (2019) Where and how centenarians die? The role of hospice care. Am J Hosp Palliat Care 36(12):1068–1075. 10.1177/1049909119845884 31035790 10.1177/1049909119845884

[28] Rabuñal Rey R, Monte Secades R, Rigueiro Veloso MT, Casariego Vales EJ, Ibáñez Alonso MD, García Pais MJ (2002) Centenarian patients attended at a general hospital. Rev Clin Esp 202(6):326–328. 10.1016/s0014-2565(02)71067-9 12093397 10.1016/s0014-2565(02)71067-9

[29] Dotchin CL, Gray WK, Gaskin E, Hartley S, Walker RW (2016) Frequency, nature and outcomes of hospital admissions in centenarians in an area of North-East England. Geriatr Gerontol Int 16(8):969–975. 10.1111/ggi.12586 26311143 10.1111/ggi.12586

[30] Twersky SE, Davey A (2022) National hospitalization trends and the role of preventable hospitalizations among centenarians in the United States (2000-2009). Int J Environ Res Public Health 19(2):795. 10.3390/ijerph19020795 35055617 10.3390/ijerph19020795 PMC8775492

[31] Martín-Sánchez FJ, Fernández-Alonso C, Hormigo AI, Jiménez-Díaz G, Roiz H, Bermejo-Boixareu C, Rodríguez-Salazar J, Fernández Pérez C, Gil-Gregorio P (2016) Clinical profile and 90-day mortality in centenarian patients attended in emergency departments. Rev Esp Geriatr Gerontol 51(4):196–200. 10.1016/j.regg.2015.12.008 26916908 10.1016/j.regg.2015.12.008

[32] Barceló M, Francia E, Romero C, Ruiz D, Casademont J, Torres OH (2018) Hip fractures in the oldest old. Comparative study of centenarians and nonagenarians and mortality risk factors. Injury 49(12):2198–2202. 10.1016/j.injury.2018.09.043 30274759 10.1016/j.injury.2018.09.043

[33] Piñeiro-Fernández JC, Rabuñal-Rey R, Romay-Lema E, Rubal-Bran D, Pedrosa-Fraga C, Santos-Martínez AM, Besteiro-Balado Y, Suárez-Gil R, Pértega-Díaz S (2025) Comorbidity burden, management, and in-hospital outcomes in centenarians with proximal hip fracture: a nationwide cohort study (2004–2020). Arch Osteoporos 20(1):88. 10.1007/s11657-025-01576-7 40646291 10.1007/s11657-025-01576-7 PMC12254154

[34] Freeman S, Armstrong JJ, Tyas SL, Neufeld E (2017) Clinical characteristics and patterns of health deficits of centenarians receiving home care and long-term care services. Exp Gerontol 99:46–52. 10.1016/j.exger.2017.09.010 28943479 10.1016/j.exger.2017.09.010

[35] Rojano I, Luque X, Sánchez Ferrin P, Salvà A (2016) Hospital complications in the elderly. Med Clin (Barc) 146(12):550–554. 10.1016/j.medcli.2015.12.015 26961393 10.1016/j.medcli.2015.12.015

[36] Evans CJ, Ho Y, Daveson BA, Hall S, Higginson IJ, Gao W, GUIDE_Care project (2014) Place and cause of death in centenarians: a population-based observational study in England, 2001 to 2010. PLoS Med 11(6):e1001653. 10.1371/journal.pmed.1001653 24892645 10.1371/journal.pmed.1001653 PMC4043499

[37] Teixeira L, Araújo L, Paúl C, Ribeiro O (2020) Further survival at age 100: findings from the Oporto Centenarian study. Exp Gerontol 133:110854. 10.1016/j.exger.2020.110854 32004634 10.1016/j.exger.2020.110854

[38] Xu J (2016) Mortality among centenarians in the United States, 2000-2014. NCHS Data Brief 233:1–8 26828422

[39] Carey JR, Judge DS (2001) Life span extension in humans is self-reinforcing: a general theory of longevity. Popul Dev Rev 27:411–436. 10.1111/j.1728-4457.2001.00411.x

[40] Gao F, Liu G, Ge Y, Tan Z, Chen Y, Peng W, Zhang J, Zhang X, He J, Wen L, Wang X, Shi Z, Hu S, Sun F, Gong Z, Sun M, Tian M, Zhu S, Yang M, Wu X (2023) Orthogeriatric co-managements lower early mortality in long-lived elderly hip fracture: a post-hoc analysis of a prospective study. BMC Geriatr 23(1):571. 10.1186/s12877-023-04289-z 37723423 10.1186/s12877-023-04289-z PMC10506232

[41] Nakanishi Y, Tsugihashi Y, Akahane M, Noda T, Nishioka Y, Myojin T, Kubo S, Higashino T, Okuda N, Robine JM, Imamura T (2021) Comparison of Japanese centenarians’ and noncentenarians’ medical expenditures in the last year of life. JAMA Netw Open 4(11):e2131884. 10.1001/jamanetworkopen.2021.31884 34739063 10.1001/jamanetworkopen.2021.31884 PMC8571656

[42] Prieto-Lara E, Ocaña-Riola R (2010) Updating rurality index for small areas in Spain. Soc Indic Res 95:267–280. 10.1007/s11205-009-9459-0

[43] Vallejo Maroto I, Cubo Romano P, Mafé Nogueroles MC, Matesanz-Fernández M, Pérez-Belmonte LM, Said Criado I, Gómez-Huelgas R, Díez Manglano J (2021) Recommendations on the comprehensive, multidimensional assessment of hospitalized elderly people. Position of the Spanish Society of Internal Medicine. Rev Clin Esp 221(6):347–358. 10.1016/j.rce.2020.10.003 34059234 10.1016/j.rceng.2020.10.007

[44] Bernabeu-Wittel M, Moreno-Gaviño L, Ollero-Baturone M, Barón-Franco B, Díez-Manglano J, Rivas-Cobas C, Murcia-Zaragoza J, Ramos-Cantos C, Fernández-Moyano A, PROFUND Researchers (2016) Validation of PROFUND prognostic index over a four-year follow-up period. Eur J Intern Med 36:20–24. 10.1016/j.ejim.2016.07.022 27491587 10.1016/j.ejim.2016.07.022

