# Supplementary material for: Trends in hospital admissions and clinical complexity in centenarians: a nationwide population-based study in Spain (2004–2020)
Source: Eur Geriatr Med. 2025 Nov 25;17(2):797–809. doi: 10.1007/s41999-025-01362-1 (PMC13109149; doi:10.1007/s41999-025-01362-1)
Supplement: Supplementary file 1 — Supplementary file1 (PDF 662 KB) [file 41999_2025_1362_MOESM1_ESM.pdf]

SUPPLEMENTARY MATERIAL

Supplementary Table 1. Demographic and clinical characteristics of centenarians hospitalized in Spain, between 2004-2020.

	**2004**	**2005**	**2006**	**2007**	**2008**	**2009**	**2010**	**2011**	**2012**	**2013**	**2014**	**2015**	**2016**	**2017**	**2018**	**2019**	**2020**	**% increase**	***p***
**Age, years**	101.8 ± 1.7	101.7 ± 1.9	101.8 ± 2.1	101.8 ± 2.3	101.6 ± 2.1	101.6 ± 2.2	101.4 ± 2.1	101.4 ± 1.9	101.4 ± 2.1	101.3 ± 1.7	101.3 ± 1.7	101.3 ± 1.6	101.3 ± 1.7	101.3 ± 1.6	101.3 ± 1.6	101.3 ± 1.6	101.3 ± 1.6	-0.5%	<0.001
**Sex, female**	69.4%	68.6%	69.4%	71.4%	72.9%	71.9%	71.8%	74.8%	73.7%	75.3%	76.5%	78.8%	77.9%	78.9%	78.6%	77.1%	77.3%	11.4%	<0.001
**Comorbidity**																			
CCI	1.2 ± 1.4	1.2 ± 1.4	1.3 ± 1.4	1.2 ± 1.4	1.4 ± 1.4	1.5 ± 1.5	1.4 ± 1.5	1.4 ± 1.5	1.4 ± 1.5	1.5 ± 1.6	1.5 ± 1.6	1.5 ± 1.6	1.7 ± 1.7	1.9 ± 1.7	2.0 ± 1.7	2.0 ± 1.8	2.1 ± 1.8	75.0%	<0.001
Severe comorbidity, CCI ≥ 3	14.6%	14.2%	15.9%	15.0%	18.8%	21.7%	20.4%	19.7%	20.9%	23.4%	23.2%	23.3%	26.3%	31.1%	33.4%	34.0%	34.3%	134.9%	<0.001
Multimorbidity	52.8%	57.9%	58.8%	60.6%	66.5%	68.8%	60.4%	63.0%	62.1%	63.6%	65.2%	64.0%	65.8%	77.3%	79.0%	81.0%	79.8%	51.1%	<0.001
Number of chronic diseases	1.9 ± 1.5	2.0 ± 1.6	2.1 ± 1.6	2.1 ± 1.6	2.3 ± 1.6	2.5 ± 1.6	2.2 ± 1.8	2.3 ± 1.7	2.2 ± 1.8	2.4 ± 1.8	2.5 ± 1.8	2.4 ± 1.8	2.5 ± 1.9	3.0 ± 1.9	3.0 ± 1.9	3.2 ± 1.9	3.2 ± 1.9	68.4%	<0.001
0	22.0%	18.5%	17.7%	18.0%	13.2%	10.3%	21.3%	18.2%	20.6%	18.4%	17.2%	18.7%	17.2%	9.1%	8.7%	7.2%	6.1%	-72.3%	
1	25.2%	23.6%	23.5%	21.4%	20.3%	20.9%	18.3%	18.8%	17.3%	18.1%	17.6%	17.3%	17.0%	13.5%	12.3%	11.8%	14.1%	-44.0%	
2	21.0%	24.4%	21.8%	23.9%	23.5%	22.3%	19.5%	18.8%	20.9%	18.5%	19.0%	17.4%	17.9%	20.2%	20.6%	19.6%	18.3%	-12.9%	
3	16.8%	16.8%	18.1%	18.3%	20.4%	21.8%	17.6%	19.9%	16.9%	16.7%	18.7%	19.0%	18.2%	20.7%	20.4%	20.5%	19.3%	14.9%	
> 3	14.9%	16.7%	18.9%	18.4%	22.6%	24.7%	23.3%	24.2%	24.3%	28.3%	27.6%	27.6%	29.6%	36.4%	38.0%	40.9%	42.2%	183.2%	
**Chronic conditions**																			
Hypertension	28.5%	32.2%	33.7%	34.7%	37.9%	40.4%	34.1%	39.2%	37.6%	35.0%	38.2%	37.1%	38.1%	43.8%	42.2%	41.8%	42.3%	48.4%	0.191
Dyslipemia	2.3%	3.0%	3.5%	3.8%	3.0%	4.0%	3.7%	5.3%	5.3%	7.3%	6.8%	7.4%	10.5%	12.0%	16.0%	19.0%	19.5%	747.8%	<0.001

Diabetes	9.5%	9.3%	11.7%	10.2%	11.9%	13.3%	13.0%	12.5%	11.9%	13.3%	13.1%	12.1%	12.5%	13.3%	14.2%	15.3%	15.1%	58.9%	0.527
CAD	10.3%	11.1%	13.1%	10.6%	11.5%	11.3%	11.1%	9.0%	9.4%	9.3%	9.1%	8.9%	9.6%	10.6%	11.7%	10.8%	11.7%	13.6%	<0.001
CHF	21.0%	21.9%	23.7%	26.0%	29.1%	27.3%	25.8%	26.5%	25.9%	27.8%	28.6%	28.5%	27.6%	33.9%	35.4%	35.4%	34.7%	65.2%	<0.001
AF	16.1%	18.9%	20.4%	20.8%	24.5%	21.2%	20.4%	21.3%	19.5%	20.9%	23.0%	21.5%	21.9%	26.3%	27.3%	27.5%	28.4%	76.4%	<0.001
Other arrhythmias	8.4%	7.7%	10.0%	9.7%	10.0%	10.1%	8.1%	7.8%	8.1%	9.0%	8.2%	7.1%	6.7%	6.5%	7.0%	7.0%	7.5%	-10.7%	0.876
COPD	9.2%	10.6%	9.9%	8.2%	11.2%	13.4%	12.2%	9.8%	9.3%	9.8%	10.5%	10.2%	9.7%	11.2%	10.8%	11.1%	10.5%	14.1%	0.537
CVD	10.0%	7.7%	7.8%	8.6%	8.3%	9.4%	11.5%	13.5%	14.0%	13.3%	14.0%	12.8%	12.8%	15.9%	15.8%	17.4%	17.5%	75.0%	<0.001
CKD	8.2%	6.5%	7.8%	9.3%	12.5%	13.5%	12.0%	13.7%	14.0%	17.1%	17.0%	18.8%	21.4%	26.4%	27.6%	28.2%	29.3%	257.3%	<0.001
Thyroid disease	2.2%	2.8%	3.1%	2.2%	2.9%	4.7%	3.1%	2.8%	3.2%	4.0%	5.0%	5.4%	5.8%	8.6%	7.3%	8.2%	9.8%	345.5%	0.017
Biliopancreatic disease	5.8%	6.8%	5.0%	6.0%	7.4%	6.9%	6.5%	6.8%	6.5%	6.4%	6.1%	5.9%	5.6%	6.4%	5.9%	6.8%	6.7%	15.5%	<0.001
Active malignant neoplasm	7.5%	7.0%	6.5%	6.0%	6.6%	7.5%	6.6%	7.0%	5.6%	6.5%	5.5%	6.3%	7.8%	5.5%	6.4%	7.1%	6.1%	-18.7%	0.931
Anemia	13.7%	14.4%	15.9%	15.9%	17.1%	18.7%	16.8%	17.6%	16.9%	20.1%	17.5%	19.1%	20.5%	24.0%	23.3%	25.3%	24.2%	76.6%	0.002
Dementia	12.9%	14.6%	14.5%	13.8%	17.6%	17.5%	13.2%	15.1%	15.0%	16.8%	16.4%	16.7%	19.5%	20.9%	21.2%	22.5%	23.7%	83.7%	<0.001
Urinary incontinence	2.3%	2.9%	3.6%	3.2%	4.4%	5.9%	3.4%	4.3%	4.1%	3.7%	4.6%	4.5%	6.3%	7.2%	7.1%	8.6%	8.5%	269.6%	<0.001
Visual loss	5.3%	5.8%	4.9%	5.5%	5.8%	7.4%	5.7%	7.1%	6.9%	6.6%	7.7%	8.7%	9.4%	9.7%	10.6%	12.7%	12.7%	139.6%	0.937
Hearing loss	7.2%	7.7%	6.5%	6.4%	8.2%	10.7%	6.5%	7.6%	8.2%	8.6%	8.2%	8.6%	8.7%	10.4%	10.3%	10.8%	12.2%	69.4%	0.024
Pressure ulcers	4.1%	4.1%	4.9%	4.8%	7.7%	5.6%	5.3%	6.7%	6.1%	5.9%	6.0%	4.7%	5.9%	6.9%	7.0%	7.1%	7.9%	92.7%	<0.001

Note: Continuous variables are expressed as mean ± standard deviation and categorical variables as percentage. Multimorbidity = ≥ 2 chronic diseases; CCI = Charlson Comorbidity index; CAD = coronary artery disease; CHF = congestive heart failure; AF = atrial fibrillation; COPD = chronic obstructive pulmonary disease; CVD = cerebrovascular disease; CKD = chronic kidney disease.

Supplementary Table 2. Changes in demographic and clinical characteristics of centenarians hospitalized in Spain, between 2004-2020, by sex.

	Male				Female			
	**2004-2010**	**2011-2015**	**2016-2020**	**% increase**	**2004-2010**	**2011-2015**	**2016-2020**	**% increase**
**Age, years**	102.2 ± 2.6 (101)	101.4 ± 2.1 (101)	101.2 ± 1.5 (101)	-1.0%	101.4 ± 1.8 (101)	101.3 ± 1.7 (101)	101.3 ± 1.6 (101)	-0.1%
**No chronic disease**	2 ± 1.6 (2)	2.3 ± 1.7 (2)	3 ± 1.9 (3)	50.0%	2.2 ± 1.6 (2)	2.4 ±1.8 (2)	3 ± 1.9 (3)	36.4%
**Multimorbidity**	56.2%	63.2%	76.1%	35.4%	63.5%	63.8%	76.8%	20.9%
**CCI**	1.4 ± 1.6 (1)	1.7 ± 1.7 (1)	2 ± 1.8 (2)	42.9%	1.3 ± 1.4 (1)	1.4 ± 1.5 (1)	1.9 ± 1.7 (2)	46.2%
**Severe comorbidity**	20.5%	27.8%	33.2%	62.0%	16.5%	20.5%	31.5%	90.9%
**Nursing home, place of residence**	10.5%	9.3%	11.7%	11.4%	12.4%	10.4%	11.8%	-4.8%
**Chronic conditions**								
Hypertension	36.5%	38.0%	36.9%	1.1%	38.7%	38.9%	39.0%	0.8%
Dyslipemia	7.4%	10.3%	10.6%	43.2%	8.1%	9.3%	10.1%	24.7%
Diabetes	12.5%	13.1%	12.6%	0.8%	13.2%	12.2%	13.3%	0.8%
CAD	9.5%	11.1%	10.7%	12.6%	9.5%	10.2%	11.3%	18.9%
CHF	27.3%	28.8%	29.2%	7.0%	28.9%	27.7%	31.6%	9.3%
AF	21.4%	21.8%	24.2%	13.1%	21.5%	22.7%	25.0%	16.3%
Other arrhythmias	7.5%	8.2%	7.8%	4.0%	8.0%	7.9%	7.8%	-2.5%
COPD	11.1%	9.8%	10.4%	-6.3%	10.3%	10.3%	10.8%	4.9%
CVD	11.4%	11.4%	15.2%	33.3%	11.5%	12.6%	15.3%	33.0%
CKD	16.6%	18.2%	20.2%	21.7%	17.2%	18.4%	20.8%	20.9%
Thyroid disease	4.8%	4.6%	5.6%	16.7%	5.3%	5.6%	5.7%	7.5%
Bilipancreatic disease	4.7%	9.1%	6.2%	31.9%	4.9%	7.6%	6.0%	22.4%
Active malignant neoplasm	6.4%	6.5%	6.1%	-4.7%	5.9%	7.6%	6.2%	5.1%
Anemia	19.4%	19.9%	20.0%	3.1%	20.5%	20.5%	19.2%	-6.3%
Dementia	15.7%	15.0%	20.1%	28.0%	16.8%	17.4%	19.9%	18.5%
Urinary incontinence	4.0%	5.9%	5.8%	45.0%	4.4%	5.8%	6.1%	38.6%
Visual loss	7.9%	8.7%	8.6%	8.9%	8.1%	8.9%	8.5%	4.9%
Hearing loss	9.9%	8.6%	8.9%	-10.1%	9.2%	9.2%	8.7%	-5.4%
Pressure ulcers	4.4%	4.6%	6.9%	56.8%	4.8%	6.7%	7.1%	47.9%

Note: Continuous variables are expressed as mean ± standard deviation (median) and categorical variables as percentage. Multimorbidity = ≥ 2 chronic diseases; CCI = Charlson Comorbidity index; CAD = coronary artery disease; CHF = congestive heart failure; AF = atrial fibrillation; COPD = chronic obstructive pulmonary disease; CVD = cerebrovascular disease; CKD = chronic kidney disease.

Supplementary Table 3. Principal diagnosis of centenarians hospitalized in Spain, between 2004-2020.

	2004	2005	2006	2007	2008	2009	2010	2011	2012	2013	2014	2015	2016	2017	2018	2019	2020	% increase	*p*
**DGR type, medical**	76.5%	80.0%	80.7%	83.7%	83.5%	84.6%	85.1%	84.1%	86.0%	86.5%	86.4%	87.4%	86.4%	88.3%	87.6%	88.7%	87.3%	14.1%	<0.001
**DGR type, surgical**	23.5%	20.0%	19.3%	16.3%	16.5%	15.4%	14.9%	15.9%	14.0%	13.5%	13.6%	12.6%	13.6%	11.7%	12.4%	11.3%	12.7%	-46.0%	
**Place of residence, nursing home**	3.8%	4.6%	6.1%	7.2%	7.1%	9.0%	7.0%	8.4%	10.5%	12.1%	12.1%	12.1%	10.7%	14.3%	13.5%	15.0%	19.4%	410.5%	0.826
**Principal diagnosis**																			
Infections, total	23.2%	26.9%	26.6%	26.0%	27.0%	29.8%	30.1%	30.2%	31.4%	32.3%	31.8%	34.1%	33.4%	39.3%	38.9%	39.0%	42.4%	82.8%	<0.001
RTI	11.2%	11.4%	12.5%	11.8%	12.5%	14.1%	14.4%	15.0%	15.7%	15.1%	14.7%	15.9%	14.0%	15.0%	14.0%	14.4%	14.4%	70.9%	<0.001
Pneumonia	3.2%	4.6%	3.5%	4.3%	4.1%	3.7%	2.6%	3.5%	3.4%	4.1%	4.6%	4.8%	6.9%	9.2%	9.8%	8.3%	6.2%	122.9%	<0.001
Acute bronchitis	2.5%	3.4%	2.6%	3.1%	2.8%	2.3%	1.4%	2.1%	2.0%	2.5%	3.0%	2.9%	2.3%	2.3%	2.8%	2.1%	0.9%	264.2%	<0.001
COPD exacerbation	2.0%	2.5%	1.8%	1.3%	1.8%	2.0%	2.1%	1.5%	1.5%	1.6%	1.7%	1.9%	1.7%	2.0%	1.5%	2.0%	1.0%	-50.0%	0.051
UTI	2.8%	3.8%	4.6%	4.1%	3.7%	4.9%	5.0%	5.7%	5.9%	5.8%	6.4%	6.5%	6.5%	7.7%	8.0%	8.3%	9.1%	225.0%	<0.001
Intraabdominal infection	2.0%	2.8%	1.6%	2.2%	2.4%	2.4%	2.9%	2.3%	2.1%	3.0%	2.0%	2.2%	2.5%	2.7%	2.5%	2.8%	3.2%	60.0%	0.010
Sepsis, without defined focus	0.7%	0.3%	0.6%	0.8%	1.2%	1.2%	1.9%	1.5%	1.7%	1.8%	1.7%	1.9%	0.6%	0.7%	0.8%	0.6%	0.6%	-14.3%	0.032
HF	9.8%	10.9%	11.0%	11.8%	12.5%	10.9%	12.4%	12.1%	12.8%	13.1%	U13.1%	13.7%	11.2%	12.2%	12.7%	12.9%	12.6%	28.6%	0.002
Hip fracture	10.7%	11.2%	12.2%	10.6%	10.3%	8.8%	9.7%	11.2%	11.0%	10.4%	10.2%	9.4%	7.8%	8.6%	9.4%	8.7%	9.8%	-8.4%	<0.001
Acute CVD	7.5%	5.6%	5.4%	5.6%	5.0%	5.8%	4.9%	5.9%	5.2%	6.0%	5.3%	4.9%	4.2%	4.6%	4.4%	4.9%	5.0%	-33.3%	<0.001
Acute Respiratory Failure	1.1%	1.3%	2.8%	4.6%	5.5%	5.9%	5.3%	5.6%	4.7%	4.2%	4.5%	4.8%	3.9%	4.0%	4.1%	3.4%	2.2%	100.0%	0.219
Fracture, other than a hip fracture	4.2%	3.0%	3.2%	3.0%	3.2%	3.0%	2.2%	2.6%	2.1%	2.8%	2.6%	2.6%	2.0%	2.2%	2.7%	2.9%	3.2%	-23.8%	0.048
Active malignant neoplasm	4.0%	3.2%	3.3%	2.7%	2.5%	2.9%	2.8%	2.9%	2.3%	2.6%	1.7%	1.9%	3.3%	1.5%	1.8%	2.0%	1.8%	-55.0%	<0.001

Gastrointestinal bleeding	2.0%	2.3%	2.2%	2.5%	2.1%	1.8%	2.7%	1.8%	2.0%	2.4%	2.5%	2.1%	2.0%	2.0%	2.2%	2.0%	1.9%	-5.0%	0.312
Acute Ischemic Heart Disease	2.8%	3.3%	3.0%	2.5%	2.2%	2.0%	1.9%	1.6%	2.3%	1.8%	2.3%	1.8%	1.6%	1.8%	1.5%	1.4%	1.0%	-64.3%	<0.001
Arrythmias	1.8%	1.3%	2.0%	2.2%	2.9%	1.6%	1.9%	2.4%	1.9%	2.1%	2.5%	1.9%	1.8%	1.6%	1.5%	1.5%	1.7%	-5.6%	0.026
Acute Arterial Occlusion	2.8%	2.2%	1.6%	2.3%	1.9%	2.2%	1.8%	2.3%	2.1%	1.9%	1.9%	1.8%	1.1%	1.8%	1.5%	1.4%	1.3%	-53.6%	<0.001
Biliopancreatic disease	1.7%	2.2%	1.2%	1.3%	1.3%	2.0%	1.4%	2.1%	2.0%	1.4%	1.9%	1.6%	1.1%	1.1%	1.3%	1.4%	1.2%	-29.4%	0.005
AKI	1.1%	1.2%	1.4%	1.0%	1.0%	1.1%	1.2%	1.7%	1.7%	1.6%	1.7%	1.5%	1.3%	1.6%	1.4%	1.7%	1.5%	36.4%	0.023
Acute gastroenteritis and enterocolitis	1.2%	1.5%	1.5%	1.2%	1.7%	1.6%	2.0%	0.7%	1.5%	1.8%	1.4%	1.4%	1.1%	1.2%	0.8%	2.1%	1.4%	16.7%	0.606
Hydroelectrolytic disorders	1.1%	1.0%	1.6%	1.3%	1.7%	1.4%	1.3%	1.1%	1.4%	1.5%	1.5%	1.4%	1.4%	1.6%	1.2%	1.1%	0.8%	-27.3%	0.176
Anemia	0.5%	0.6%	1.0%	1.2%	0.7%	0.8%	1.7%	1.6%	0.8%	1.6%	0.9%	1.2%	1.6%	1.2%	1.1%	0.9%	1.0%	100.0%	0.281

Note: DGR = Diagnosis-related group; RTI = Respiratory Tract Infection; COPD = chronic obstructive pulmonary disease; UTI = Urinary tract infection; CVD = cerebrovascular disease; HF = heart failure; AF = atrial fibrillation; AKI = acute kidney injury.

Supplementary Table 4. Changes in principal diagnosis of centenarians hospitalized in Spain, between 2004-2020, by sex.

	**Male**				**Female**			
	**2004-2010**	**2011-2015**	**2016-2020**	**% increase**	**2004-2010**	**2011-2015**	**2016-2020**	**% increase**
**DGR type, surgical**	17.3%	11.0%	10.2%	-41.0%	18.0%	14.7%	12.9%	-28.3%
**Principal diagnosis**	82.7%	89.0%	89.8%	8.6%	82.0%	85.3%	87.1%	6.2%
Infections, total	28.9%	37.4%	39.9%	38.1%	26.8%	30.4%	38.2%	42.5%
RTI	20.2%	24.9%	27.7%	37.1%	17.7%	20.1%	25.6%	44.6%
Pneumonia	14.4%	18.2%	16.0%	11.1%	12.1%	14.4%	13.9%	14.9%
Acute bronchitis	2.7%	3.6%	7.7%	185.2%	4.1%	4.3%	8.2%	100%
COPD exacerbation	3.1%	3.0%	2.1%	-32.3%	1.5%	1.3%	1.5%	0.0%
UTI	4.2%	7.2%	7.8%	85.7%	4.2%	5.7%	8.0%	90.5%
Intraabdominal infection	1.9%	2.4%	2.6%	36.8%	2.5%	2.3%	2.8%	12.0%
Sepsis, without defined focus	1.0%	2.2%	0.6%	-40.0%	1.0%	1.6%	0.7%	-30.0%
HF	9.3%	11.0%	12.3%	32.3%	12.3%	13.6%	12.3%	0.0%
Hip fracture	6.6%	6.7%	7.5%	13.6%	11.9%	11.5%	9.3%	-21.8%
Acute CVD	5.0%	5.1%	4.6%	-8.0%	5.9%	5.5%	4.6%	-22.0%
Acute Respiratory Failure	3.6%	4.7%	3.8%	5.6%	4.2%	4.8%	3.5%	-16.7%
Fracture, other than a hip fracture	2.8%	1.4%	2.1%	-25.0%	3.1%	2.9%	2.7%	-12.9%
Active malignant neoplasm	4.3%	3.3%	2.4%	-44.2%	2.8%	2.1%	2.2%	-21.4%
Gastrointestinal bleeding	2.3%	2.3%	1.9%	-17.4%	2.2%	2.1%	2.0%	-9.1%
Acute Ischemic Heart Disease	2.7%	2.2%	1.6%	-40.7%	2.4%	1.9%	1.4%	-41.7%
Arrhythmias	2.0%	2.3%	1.3%	-35.0%	2.0%	2.1%	1.7%	-15.0%
Acute Arterial Occlusion	1.8%	1.6%	1.3%	-27.8%	2.2%	2.1%	1.5%	-31.8%
Biliopancreatic disease	1.2%	1.7%	1.3%	8.3%	1.8%	1.8%	1.2%	-33.3%
AKI	1.8%	2.3%	2.0%	11.1%	0.9%	1.4%	1.4%	55.6%
Acute gastroenteritis and enterocolitis	1.4%	1.2%	1.4%	0.0%	1.6%	1.4%	1.3%	-18.8%
Hydroelectrolytic disorders	1.3%	1.3%	1.1%	-15.4%	1.4%	1.4%	1.2%	-14.3%
Anemia	0.9%	1.1%	1.1%	22.2%	1.0%	1.3%	1.1%	10.0%

Note: DGR = Diagnosis-related group; RTI = Respiratory Tract Infection; COPD = chronic obstructive pulmonary disease; UTI = Urinary tract infection; CVD = cerebrovascular disease; HF = heart failure; AF = atrial fibrillation; AKI = acute kidney injury. * The number of hospital admissions for SARS-CoV-2 in 2020 was 211 (1.2% of total admissions between 2016 and 2020), and the percentage of total RTI between 2016 and 2020 excluding SARS-CoV-2 hospital admissions was 24%.

Supplementary Table 5. Outcomes related to hospitalization of centenarians in Spain, between 2004-2020.

	**2004**	**2005**	**2006**	**2007**	**2008**	**2009**	**2010**	**2011**	**2012**	**2013**	**2014**	**2015**	**2016**	**2017**	**2018**	**2019**	**2020**	**% increase**	***p***
**In-hospital complications**	1,373	1,447	1,516	1,705	1,786	2,002	2,209	2,356	2,506	2,626	2,779	3,241	3,566	3,537	3,854	3,773	3,454	151.6%	
Acute respiratory failure	15.6%	17.4%	16.6%	16.7%	20.0%	20.2%	15.8%	16.1%	18.0%	20.2%	17.7%	18.9%	20.3%	25.9%	24.2%	24.4%	24.8%	59.0%	<0.001
AKI	5.0%	6.0%	6.7%	6.2%	8.0%	9.0%	8.1%	10.9%	11.2%	12.4%	12.4%	12.3%	14.5%	18.6%	18.4%	18.6%	20.5%	310.0%	<0.001
Hydroelectrolytic disorders	7.9%	9.7%	7.8%	9.6%	12.5%	12.6%	9.3%	10.0%	10.5%	10.9%	10.8%	11.7%	13.7%	16.3%	15.5%	15.7%	18.9%	139.2%	<0.001
Functional gastrointestinal disorders	3.6%	4.0%	4.4%	3.5%	4.1%	5.1%	5.2%	4.8%	6.7%	6.5%	6.9%	5.7%	6.9%	8.2%	7.5%	8.4%	9.5%	163.9%	<0.001
Anemia	13.2%	13.9%	14.9%	14.7%	16.4%	17.9%	15.1%	16.0%	16.1%	18.5%	16.6%	17.9%	18.9%	22.8%	22.3%	24.4%	23.2%	75.8%	<0.001
Malnutrition	2.6%	4.2%	4.4%	4.4%	4.7%	5.2%	3.9%	4.5%	4.7%	5.4%	4.8%	6.2%	7.7%	10.3%	11.9%	11.9%	13.4%	415.4%	<0.001
Delirium	6.8%	5.7%	5.1%	5.6%	5.6%	6.3%	4.8%	5.3%	5.1%	5.6%	5.6%	6.3%	6.7%	7.8%	9.4%	8.5%	9.4%	38.2%	<0.001
Pressure ulcers	3.7%	3.7%	4.6%	4.4%	6.7%	5.2%	4.6%	5.9%	5.7%	5.6%	5.4%	4.3%	5.2%	6.4%	6.5%	6.2%	7.2%	94.6%	<0.001
UTI	5.4%	5.7%	7.8%	6.5%	8.4%	9.4%	6.1%	7.0%	6.9%	7.2%	7.3%	7.9%	8.6%	10.1%	9.8%	11.1%	12.9%	138.9%	<0.001
RTI	5.9%	5.4%	6.7%	7.1%	7.7%	7.8%	2.9%	3.9%	3.6%	4.0%	3.6%	4.4%	5.2%	6.2%	7.4%	7.2%	6.2%	5.1%	0.058
Sepsis	1.0%	1.1%	1.6%	2.1%	2.4%	3.0%	2.1%	2.2%	2.6%	2.7%	2.6%	3.5%	3.5%	4.8%	5.1%	4.5%	4.4%	340.0%	<0.001
Other infections	6.4%	6.1%	6.9%	5.2%	8.0%	7.9%	6.4%	6.9%	7.3%	7.7%	8.4%	8.2%	8.9%	10.5%	10.7%	12.8%	22.0%	243.8%	<0.001
**No. in-hospital complications**																			
None	51.9%	48.6%	46.9%	47.2%	40.2%	39.1%	51.1%	48.7%	47.2%	43.7%	45.9%	44.7%	41.6%	30.7%	30.3%	29.3%	25%	-51.8%	<0.001
1	26.9%	29.2%	29.4%	29.8%	29.6%	29.1%	25%	24.5%	24.1%	26.1%	23.3%	23.4%	23.4%	26.3%	26.1%	25.4%	22.9%	-14.9%	
2	11.5%	11.4%	11.3%	12.1%	14.6%	15.6%	11.7%	12.1%	13.7%	13.4%	14.5%	14.7%	15.3%	17.9%	18%	18%	15.1%	31.3%	
≥ 3	9.6%	10.9%	12.5%	10.9%	15.6%	16.2%	12.2%	14.7%	15%	16.7%	16.2%	17.2%	19.7%	25,1%	25.6%	27.3%	37%	285.4%	
**In-hospital mortality**	21.8%	23.4%	24.1%	23.2%	24.6%	24.7%	23.3%	25.2%	25.1%	26.0%	23.4%	26.0%	22.8%	26.4%	27.0%	26.5%	27.9%	28.0%	<0.001

**LOS, days**	8.7 ± 9.5	8.8 ± 8.7	9.1 ± 14.3	9.2 ± 9.2	9.1 ± 10.7	8.2 ± 7.8	8.3 ± 11.6	8.2 ± 8.9	7.9 ± 9.1	7.3 ± 6.5	7.5 ± 6.5	7.6 ± 7.4	6.7 ± 6.4	7.5 ± 7.0	7.3 ± 6.5	7.3 ± 8.3	7.7 ± 7.9	-11.5%	<0.001
**Place of discharge, nursing home**	0.9%	0.3%	2.2%	2.8%	2.8%	2.3%	2.8%	2.5%	3.0%	3.2%	4.0%	4.8%	4.5%	4.7%	4.9%	5.1%	4.5%	400.0%	<0.001

Note: Continuous variables are expressed as mean ± standard deviation and categorical variables as percentage. AKI = Acute Kidney Injury; UTI = Urinary tract infection; RTI = Respiratory Tract Infection; LOS = Length Of hospital Stay.

Supplementary Table 6. Sex-Based Trends in In-Hospital Outcomes of Centenarians Hospitalized in Spain (2004–2020). (a) % patients with 3 or more in-hospital complications, (b) %patients with more than a week of length of stay, (c) %in-hospital mortality.

																				Trend 1		Trend 2	
		**2004**	**2005**	**2006**	**2007**	**2008**	**2009**	**2010**	**2011**	**2012**	**2013**	**2014**	**2015**	**2016**	**2017**	**2018**	**2019**	**2020**	**% increase**	**Years**	**APC (95% CI)**	**Years**	**APC (95% CI)**
**In-hospital mortality**	Total	21,8%	23,4%	24,1%	23,2%	24,6%	24,7%	23,3%	25,2%	25,1%	26,0%	23,4%	26,0%	22,8%	26,4%	27,0%	26,5%	27,9%	28,0%	2004-2022	1,0 (0,5;1,6)*		
	Females	23,8%	24,3%	24,6%	22,9%	24,2%	25,3%	23,9%	25,6%	24,7%	25,4%	22,9%	25,7%	22,1%	25,9%	26,9%	26,1%	27,1%	13,9%	2004-2021	0,7 (0,1;1,3)*		
	Males	17,1%	21,4%	22,8%	23,8%	25,6%	23,3%	21,5%	24,1%	26,0%	27,6%	25,0%	27,0%	25,3%	28,0%	27,1%	27,9%	30,4%	77,8%	2004-2020	2,1 (1,3;2,8)*		
**LOS more than 7 days**	Total	41,2%	43,5%	43,7%	45,9%	44,1%	40,5%	41,3%	41,5%	40,4%	36,6%	39,7%	38,3%	34,3%	37,2%	38,1%	34,6%	38,6%	-6,3%	2004-2022	-1,3 (-1,9 ; -0,8)*		
	Females	42,0%	43,4%	43,1%	46,1%	45,0%	39,8%	42,2%	42,6%	40,3%	36,6%	39,1%	37,4%	34,2%	36,7%	37,7%	34,0%	39,0%	-7,1%	2004-2021	-1,4 (-2,0 ; -0,8)*		
	Males	39,5%	43,6%	45,0%	45,2%	41,7%	42,3%	39,1%	38,4%	40,6%	36,6%	41,9%	41,5%	34,5%	38,9%	39,6%	36,7%	37,2%	-5,8%	2004-2020	-1,0 (-1,6 ; -0,4)*		
**>=3 in-hospital complications**	Total	9,6%	10,9%	12,5%	10,9%	15,6%	16,2%	12,2%	14,7%	15,0%	16,7%	16,2%	17,2%	19,7%	25,1%	25,6%	27,3%	37,0%	285,4%	2004-2015	4,2 (1,1;7,3)*	2015-2020	14,9 (9,7;20,4)*
	Females	9,5%	11,0%	13,5%	10,8%	15,1%	15,7%	11,8%	14,6%	14,8%	16,3%	15,4%	17,1%	19,6%	25,2%	25,3%	27,6%	37,7%	296,8%	2004-2015	3,9 (0,7;7,2)*	2015-2020	15,9 (10,7;21,5)*
	Males	9,8%	10,6%	10,1%	11,3%	16,9%	17,4%	13,0%	15,0%	15,5%	17,9%	19,0%	17,9%	19,9%	24,5%	26,6%	26,2%	34,6%	253,1%	2004-2020	7,4 (5,9;9,0)*		

Note: APC = Average Percentage Change; CI = Confidence Interval; LOS = Length Of hospital Stay.
